# Antioxidative and chaperone‐like activities of a bacterioruberin‐rich extract: An innovative approach to protect the skin proteome

**DOI:** 10.1111/ics.70032

**Published:** 2025-10-21

**Authors:** Julie C. Tisserand, François‐Xavier Pellay, Nicolas Lecland, Arnaud Fontbonne, Félix Giraud, Eric Perrier, Sandra Trompezinski, Isabelle Benoit

**Affiliations:** ^1^ Institut NAOS Aix‐En‐Provence France; ^2^ NAOS Ecobiology Company (Bioderma, Institut Esthederm, Etat Pur) Aix‐En‐Provence France

**Keywords:** bacterioruberin, chaperone, ecobiology, oxidative stress, proteome, skin

## Abstract

**Objective:**

Oxidative stress and its induced protein alterations are instrumental in the early onset and progression of ageing. To protect the skin proteome, we evaluated the extract of a bacterium isolated from snowflakes (*Arthrobacter agilis*). This *Arthrobacter agilis* extract (AAE) has been found to be rich in bacterioruberins, C‐50 unsaturated carotenoids with potent antioxidative properties.

**Methods:**

The *Arthrobacter agilis* extract (AAE), having protective effect against oxidative, saline, and heat stresses, was evaluated in tubo. Protection against protein carbonylation was assessed in human primary keratinocytes and skin explants subjected to various stresses. The impact of an AAE‐containing cream on protein carbonylation was analysed on the face of 23 smokers after 28 days.

**Results:**

In tubo, AAE protects alkaline phosphatase against oxidative and heat stresses, increasing the temperature at which BSA is denatured. It also partially prevented elastin aggregation induced by a salt stress. In human primary keratinocytes exposed to UV, particulate matter (PM), or blue light, AAE reduced protein carbonylation, a marker of oxidative stress in the proteome. When formulated in creams, topical applications prevent protein carbonylation in the epidermis and dermis of skin explants co‐exposed to UV and PM. Furthermore, after 28 days of application, protein carbonylation was reduced in the upper skin layers of smokers.

**Conclusions:**

AAE protects the proteome against oxidative stress via a dual mode of action: antioxidant and chaperone‐like activities (as demonstrated by protection against heat and salt). AAE is a promising age‐management compound that safeguards the fragile skin ecosystem in an ecobiological approach, protecting the effectors of healthy skin functioning and reinforcing natural defences when overwhelmed.

## Graphical abstract

The bacteriorubin‐rich extract of *Arthrobacter agilis* has high antioxidative properties, protecting proteins against oxidative stress and preventing their carbonylation. It also protects proteins against saline and heat stresses, suggesting a dual mode of action including antioxidant and chaperone‐like activities.

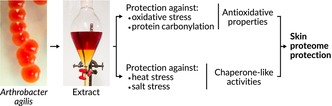

## Résumé

**Objectif:**

Le stress oxydatif et les altérations protéiques induites jouent un rôle dans l'apparition précoce et la progression du vieillissement. Pour protéger le protéome de la peau, nous avons évalué un extrait d'une bactérie isolée de flocons de neige (*Arthrobacter agilis*). Cet extrait d'*Arthrobacter agilis* (AAE) s'est révélé riche en bactériorubérines, des caroténoïdes insaturés en C‐50 aux puissantes propriétés antioxydantes.

**Méthodes:**

L'effet protecteur de l'extrait d'*Arthrobacter agilis* (AAE) contre les stress oxydatif et salin a été évalué in tubo. La protection contre la carbonylation des protéines a été évaluée dans des kératinocytes primaires humains et des explants de peau soumis à divers stress. L'impact d'une crème contenant l'AAE sur la carbonylation des protéines a été analysé sur le visage de 23 fumeurs après 28 jours.

**Résultats:**

In tubo, l'AAE protège la phosphatase alcaline contre les stress oxydatifs et thermiques, augmentant la température de dénaturation de l'albumine sérique bovine. Il prévient aussi partiellement l'agrégation de l'élastine induite par un stress salin. Dans les kératinocytes primaires humains exposés aux UV, aux particules (PM) ou à la lumière bleue, l'AAE réduit la carbonylation des protéines, un marqueur du stress oxydatif du protéome. L'application topique d'une crème contenant l'AAE prévient la carbonylation des protéines de l'épiderme et du derme d'explants cutanés co‐exposés aux UV et aux particules. En outre, après 28 jours d'application, la carbonylation des protéines a été réduite dans les couches supérieures de la peau des fumeurs.

**Conclusions:**

L'AAE protège le protéome contre le stress oxydatif grâce à un double mode d'action: une activité antioxydante et une activité de type chaperon (comme le démontre la protection contre la chaleur et le sel). L'AAE est un composé prometteur pour limiter les effets du vieillissement. Par une approche écobiologique, il protège l'écosystème fragile de la peau, les effecteurs d'un fonctionnement sain de la peau et renforçe les défenses naturelles lorsqu'elles sont débordées.

## INTRODUCTION

Aged skin is characterized by wrinkles, laxity, elastosis, aberrant pigmentation, fragility, xerosis, reduced healing, and a higher susceptibility to infections or diseases. These signs result from the combination of two processes [1]. The first is intrinsic ageing, which is the physiological and genetically programmed senescence of cells. The second, extrinsic ageing, results from interactions with environmental stressors, such as UV, pollutants, and blue light. These processes are highly interdependent [2], collectively contributing to the ageing process by affecting the same targets via common mechanisms, primarily the oxidative stress caused by reactive oxygen species (ROS) [3, 4].

ROS play an important role in the normal regulation of various signalling pathways and cellular functions [5]. However, when ROS levels exceed the capacity of the endogenous neutralizing systems, they can lead to lipid peroxidation, DNA damage, and protein alterations [6]. DNA damage is considered essential [7]. However, protein alterations play a crucial role, allowing for the consideration of proteome protection as one of the most comprehensive strategies for preventing ageing [8, 9, 10, 11]. While the oxidation of protein backbones can lead to their fragmentation, reactions with amino acid side chains trigger irreversible covalent modification, frequently carbonylation, which is an alteration specific to proteins [12, 13, 14, 15]. When these reactions functionally or structurally affect important residues, they can lead to the loss of protein function and/or alter secondary structures with exposed hydrophobic surfaces, resulting in non‐specific protein–protein interactions and aggregate formation [16, 17]. These aggregates impair the activity of cellular proteolytic systems, causing further accumulation of oxidized proteins and impaired cellular function, which can induce apoptotic cell death and/or age‐related diseases [16, 17]. Besides, peroxidation of polyunsaturated lipids generates highly reactive carbonyl species that contribute to oxidative damage [18].

Several intrinsic mechanisms counteract oxidative stress. Apart from enzymes (superoxide dismutase, catalase, glutathione peroxidase, and glutathione reductase), cells rely on various compounds to prevent or repair oxidative damages and inactivate ROS [19, 20]. However, these defences can be overwhelmed, and the classical preventive approach is to reinforce them [21, 22]. This is achieved using systemic compounds, such as vitamins C or E, carotenoids, and/or trace elements (copper or selenium). Topical application of small‐molecular‐weight antioxidants, such as vitamins (C, B3, or E), carotenoids, or polyphenols, is also possible. However, maintenance of the conformational integrity of the proteome also involves molecular chaperones. In addition to accurately folding *de novo* synthesized complex proteins, chaperones also prevent the aggregation of stress‐denatured proteins, thus enabling the rapid recovery of cellular mechanisms after stress [23, 24, 25, 26].

A proteome protection strategy could consist of an ecobiological approach that preserves the fragile skin ecosystem, promotes healthy functioning, and reinforces natural defences [27, 28]. In skin ageing, preserving the proteome and its function is essential for maintaining skin homeostasis and managing ageing. Doing so relies on biomimicry. Extremophilic bacteria have developed adaptive mechanisms that enable them to survive under conditions close to the physical and chemical limits of life, including extreme oxidative stress [29]. One such mechanism is the synthesis of bacterioruberin and its glycosylated derivatives by archaeal halophilic microorganisms [30]. Bacterioruberin is an intense red dipolar C50 isoprenoid with 13 conjugated double bonds and four terminal hydroxyl groups. With their long carbon chains, bacterioruberins have an unusually high number of double bonds, making them potent antioxidants [31]. Apart from this antioxidative activity, they also regulate membrane fluidity and permeability, and contribute to salt and heat resistance [30, 31, 32]. However, these properties are insufficient to explain the protective capacity of bacterioruberins whose activities and potential remain largely unexplored.

We used a snowfall isolate of *Arthrobacter agilis*, a bacterial species resistant to various stresses [33], and assessed the beneficial effects of a bacterioruberin‐rich extract. This *Arthrobacter agilis* extract (AAE) produced from a bacterium isolated from snowflakes was evaluated in vitro, ex vivo, and in vivo, focusing on skin proteome protection. In addition to its antioxidant properties, it also reduces protein sensitivity to heat and salt stress in tubo, possibly via a chaperone‐like mechanism. This two‐pronged biological and physical mechanism prevents age‐induced alterations in the skin proteome.

## MATERIALS AND METHODS

### *Arthrobacter agilis* extract (AAE)

The extract analysed in this study was obtained from an *Arthrobacter agilis* strain isolated from snowfall in Paris. Using this strain, an intense red, carotenoid‐rich extract was produced using a proprietary biotechnological process. Characterization indicated that it is rich in bacterioruberin, a C‐50 carotenoid with 13 unsaturated dienes, and several of its derivatives, including glycosylated ones. Because of its low water solubility, stock solutions of AAE containing 5% DMSO were used for in vitro experiments. A corresponding DMSO dose was used as a control.

For ex vivo skin explant experiments and clinical studies, AAE was topically applied in a cream and compared with a placebo cream. To quantify stress‐induced total protein carbonylation and elastin degradation in ex vivo skin explants, the effects of the placebo cream were compared with those of cream supplemented with 0.25% (w/w) tocopherol or 0.1% (w/w) AAE. In the clinical study, the effects of a placebo cream were compared with those of a cream supplemented with AAE.

### Alkaline phosphatase protection test against heat and oxidative stresses

The alkaline phosphatase activity test quantified the capacity of the compounds to protect the enzyme from different stresses. To do so, 5.7 × 10^−3^ DEA units of bovine alkaline phosphatase (P0114, Sigma‐Aldrich) were supplemented with different protective compounds, including AAE. After the addition of oxidant (H_2_O_2_, 0.75%, and FeSO_4_, 2 × 10^−4^ mol/L to produce hydroxide for 15 min at 37°C) or heat stress (1 h at 55°C), the p‐nitrophenylphosphate alkaline phosphatase substrate (P7998, Sigma‐Aldrich) was added. Reaction kinetics were measured at 405 nm for 20 min at 37°C.

Each experimental series included a negative control without a protective compound and a positive control without oxidative or heat stress. The protective activity of AAE was compared with that of known references or similar compounds.

### In tubo quantification of the stress‐induced aggregation of elastin

Aggregation of purified elastin (200 × 10^−6^ mol/L, E6527, Merck) was induced using various concentrations of NaCl, supplemented (or not) with AAE (3 × 10^−6^ mol/L). Aggregation was quantified by turbidimetry at 400 nm within a minute after adding NaCl solution [34].

### Assessment of AAE‐protein interaction

The interaction between AAE and proteins, as well as its functional consequences, was evaluated using nano differential scanning fluorimetry. Experiments were performed with a Prometheus NT.Plex instrument, gradually increasing the temperature of a bovine serum albumin (BSA) solution from 15°C to 95°C. Conformational changes were monitored by measuring the fluorescence emission ratio at 330 and 350 nm, which primarily reflect the microenvironment of tryptophan residues. Data analysis was based on the first derivative of the F330/F350 ratio with respect to temperature (δ(F330/F350)/δT), comparing a control BSA solution (25 × 10^−6^ mol/L) with a BSA solution supplemented with 0.02% AAE. 4‐phenyl butyrate (100 × 10^−6^ mol/L), a known chemical chaperone [35], was used as a positive control.

### Quantification of stress‐induced total protein carbonylation in human primary keratinocytes

Human primary keratinocytes (passage 3) were cultured in SFM (Gibco) and replaced daily. On day 1, AAE was added (treated control), or not (untreated control), to the medium. On day 2, keratinocytes were subjected to stress (blue light: 460 nm, 72 J/cm^2^ for 20 min, UVA: 365 nm, 28 J/cm^2^ for 5 min, or a small‐particle organic pollutant: PM_10_‐like, 50 mg/L, ERM‐CZ100, Merck) or left as unstressed control. After irradiation or 24 h of incubation with the small particles, the keratinocytes were fixed in 95% ethanol and 5% acetic acid. Carbonylated proteins were labelled with Oxi‐DIGE fluorescent probes [36, 37], whereas total proteins were labelled with a Cy5 NHS ester (Lumiprobe). Fluorescence was quantified with a Leica DMi8 epifluorescence microscope using ImageJ software. The results are expressed as the percentage of protein carbonylation compared with the unstressed, unprotected control.

### Quantification of stress‐induced total protein carbonylation and elastin degradation in ex vivo skin explants

Abdominal skin explants were obtained after receiving informed consent from a 30‐year‐old male subject. The DMEM culture medium was renewed daily, and explants received 2 mg/cm^2^ of topically applied cream, either a placebo cream, a 0.1% AAE‐containing cream, or a 0.25% tocopherol cream. After 4 days, explants were subjected to topical application of PM_10_‐like pollutants (30 μg, ERM‐CZ100, Merck) and UVA irradiation (460 nm, 6 J/cm^2^). Four hours after stress, explants were embedded in OCT, snap‐frozen, and cut into 4 μm‐thick sections that were fixed in 95% ethanol and 5% acetic acid. Part of the explant sections was labelled with Oxi‐DIGE fluorescent probes [36, 37] to quantify total carbonylated proteins and counterlabelled with DAPI. Non‐specific saturation with 5% BSA was applied to the remaining sections before labelling with rabbit anti‐elastin primary antibodies (sc‐25 736, Santa Cruz Biotechnology) and goat anti‐rabbit secondary antibody coupled to Alexa Fluor 488 (A11008, Invitrogen). Labelling was performed using a Leica DMi8 epifluorescence microscope under identical conditions and analysed using the ImageJ software. The results were expressed as a percentage of protection.

### In vivo quantification of protein carbonylation

This was a non‐invasive cosmetic study conducted in Poland. Local and EU regulations require no approval by an ethics committee. The principles of the Declaration of Helsinki were adhered to. All participants received detailed information and provided written informed consent before participating.

Twenty‐three subjects (aged 38–69 years, mean ± SD: 56 ± 2) who were smokers were recruited. The effect of AAE‐containing cream on protein carbonylation was compared with that of the placebo cream. Each cream was applied to a randomly selected hemiface every morning and evening. On day 0, before cream application, and after 28 days, cheekbone skin was sampled using D'Squame® discs. After extraction of proteins with the OxiProteomics® extraction buffer, carbonylated proteins were labelled with Oxi‐DIGE fluorescent probes [36, 37], separated by electrophoresis on a 4%–20% gradient SDS‐PAGE gel, and total proteins were labelled in‐gel with SyproRuby™ (Life Technologies). Carbonylated proteins were detected by fluorescence scanning at 650 nm, whereas total proteins were detected by differential fluorescence at 595 nm. Digital gel images were analysed using ImageJ software. The results are expressed as normalized carbonyl levels, that is, the signal of carbonylated proteins normalized to that of the total proteins in each sample.

### Statistical analysis

Means and standard deviations were calculated for all experiments. Additionally, the percentages of variation versus a reference, either the untreated, unstressed control, or a placebo group, were calculated. Subsequently, the means and standard deviations of these percentages were calculated.

All statistical comparisons were performed using raw data. After checking for normal data distribution using a Shapiro–Wilk test, results were compared using a two‐tailed paired Student's t‐test for the NaCl‐induced aggregation of elastin, an ANOVA followed by Bonferroni's multiple comparison tests for primary carbonylation in keratinocytes, or Mann–Whitney tests or Dunnett's multiple comparison tests for all results from ex vivo skin explants. In vivo quantification of protein carbonylation was analysed using the Friedman test followed by pairwise Wilcoxon sum rank test.

## RESULTS

### In tubo, AAE protects alkaline phosphatase against oxidative and heat stresses

**FIGURE 1:**
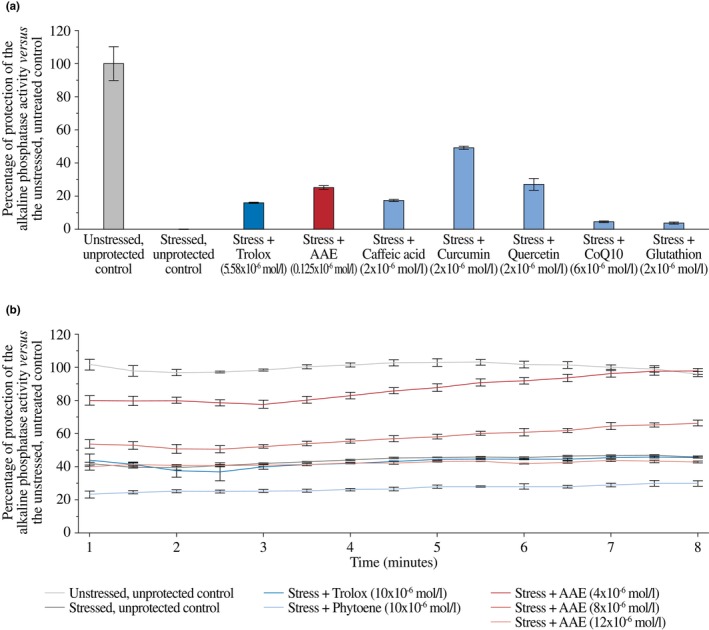
Protection of the alkaline phosphatase activity against stresses. (a) Protection against oxidative stress. (b) Protection against heat stress. Results are from duplicates and expressed as the percentage of protection compared with the unstressed, untreated control. For oxidative stress, protection was assessed based on alkaline phosphatase activity measured after 9 min. For heat stress, protection was quantified by monitoring enzyme activity between two time points separated by 30 s.

To assess the protective properties of AAE, we evaluated its capacity to protect alkaline phosphatase activity under oxidative conditions (Figure 1a). All antioxidants tested resulted in some protection (4–49%) when used at micromolar concentrations (2–6 × 10^−6^ mol/L), but AAE led to a 16% protection when used at a much lower concentration (0.125 × 10^−6^ mol/L).

To determine whether AAE also protects alkaline phosphatase against other stresses, we evaluated the effect of heat stress (Figure 1b). Neither phytoene, a carotenoid precursor, nor Trolox had any protective effects, showing only a residual activity that is stable throughout the experiment. In contrast, AAE was the only compound conferring protection. Between 30 s and 1 min after AAE addition, alkaline phosphatase activity reached 53.7% of that of the unstressed, unprotected control following the addition of 8 × 10^−6^ mol/L of AAE, and increased to 80.1% with 12 × 10^−6^ mol/L of AAE. Besides this rapid, dose‐dependent effect, AAE progressively enhanced alkaline protection over time, resulting in an activity of 66.4% for 8 × 10^−6^ mol/L of AAE and 97.9% upon the addition of 12 × 10^−6^ mol/L, a latter value similar to that of the unstressed, unprotected control.

### AAE prevents stress‐induced aggregation of elastin in tubo

**FIGURE 2:**
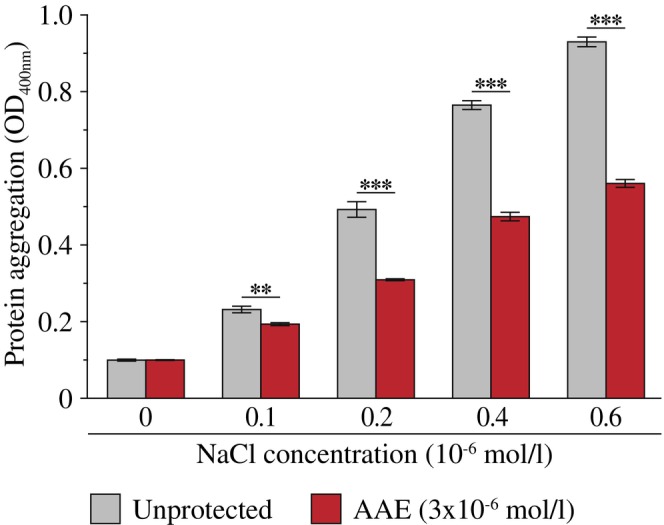
Prevention of elastin aggregation upon NaCl stress. Results are from triplicate experiments with **p < 0.01, and ***p < 0.001.

The next step was to determine, in tubo, whether AAE could prevent extracellular matrix protein aggregation. Focusing on elastin subjected to increasing concentrations of sodium chloride (NaCl), AAE supplementation partially prevented its aggregation (Figure 2).

### AAE interacts with proteins to protect them from heat‐induced denaturation

To determine whether AAE interacts with proteins and assess the effect of its supplementation, we monitored heat‐induced conformational changes of BSA using nano differential scanning fluorimetry.

**FIGURE 3:**
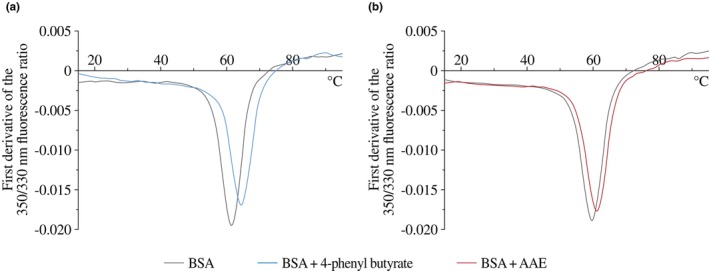
First derivative of the 330/350 nm fluorescence ratio with respect to temperature for (a) BSA and BSA supplemented with 4‐phenyl butyrate and (b) BSA and BSA supplemented with AAE.

Analysis of the first derivative of the fluorescence emission ratio at 330/350 nm with respect to temperature (Figure 3) showed that supplementation with 4‐phenyl butyrate, a well‐established chemical chaperone, increased the temperature of conformational transition by 2.9°C compared with BSA alone. Similarly, AAE supplementation shifted the transition temperature by +1.4°C, indicating a stabilizing effect upon heat‐induced denaturation.

### AAE prevents stress‐induced carbonylation of proteins in human primary keratinocytes

The protective effects of AAE against protein carbonylation were evaluated in human primary keratinocytes exposed to particulate matters (PM), UVA, or blue light. Relative quantification (Figure 3) indicated that all stresses induced a significant increase in carbonylation, and that whatever stress was considered, this increase was largely and significantly reduced upon the addition of AAE.

### AAE prevents stress‐induced carbonylation of proteins and elastin degradation in ex vivo skin explants

**FIGURE 4:**
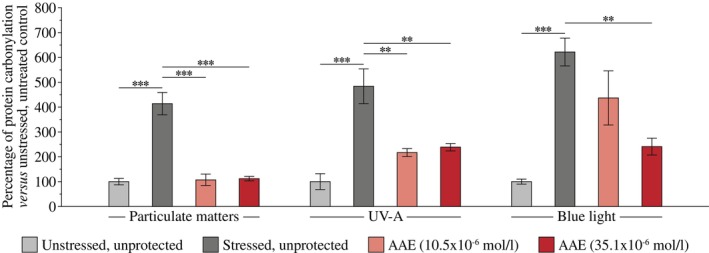
Prevention of stress‐induced protein carbonylation in human primary keratinocytes. Results are from three replicates with primary keratinocytes from one donor. They are expressed as the percentage of the unstressed, untreated control with **p < 0.01, and ***p < 0.001.

When analysing protein carbonylation in ex vivo skin explants, UV irradiation in the presence of PM increased the levels of carbonylated proteins (Figure 4a–c). As expected, this increase was more pronounced in the papillary dermis (Figure 4c). Topical application of a cream containing tocopherol or AAE reduced the overall protein carbonylation compared with stressed, unprotected control and stressed, placebo‐treated skin explants. Besides, it should be noted that AAE provided better protein protection compared with tocopherol even though present at a lower concentration. Furthermore, while there was little difference between the two compounds in the epidermis, only AAE had a significant protective effect on the dermis (Figure 4a,b).

UV and PM stress decreased elastin labelling compared with the unstressed, untreated control (Figure 4d). The topical application of the tocopherol‐containing cream did not protect elastin better than the placebo cream, whereas the AAE‐containing cream did. This effect was significant compared with the stressed, unprotected control and stressed, placebo‐treated skin explants.

### Topical application of a cosmetic cream containing AAE reduces protein carbonylation in vivo

**FIGURE 5:**
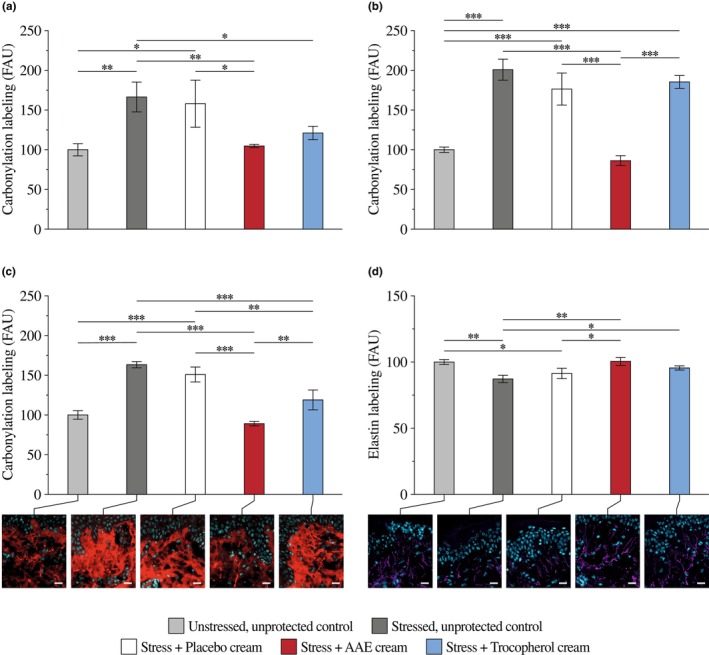
Relative quantification of immunofluorescent labelling from protein carbonylation and elastin in skin explants. (a) Quantification of protein carbonylation in the epidermis. (b) Quantification of protein carbonylation in the dermis. (c) Quantification of total protein carbonylation and representative immunofluorescence images. (d) Quantification of elastin and representative immunofluorescence images. Results are from the analysis of three images per condition using skin explants from the same donor. They are expressed as the percentage of fluorescent signal compared with the unstressed, unprotected control. Only relevant statistical differences versus the stressed, unprotected control and the stressed, placebo‐treated explant are reported with *p < 0.5, **p < 0.01, and ***p < 0.001.

Finally, we evaluated the effects of AAE on the levels of carbonylated proteins in the superficial skin of smokers. We compared the effect of a cosmetic cream containing AAE with that of a placebo, each applied twice daily to the hemiface for 28 days (Figure 5).

Normalized carbonyl levels (Table 1) indicated that the hemifaces to which the placebo was applied showed no significant variation over the 28 days (*p* = 0.893). Conversely, in the hemifaces that received the AAE‐containing cream, the level of carbonylated protein significantly decreased by 17% within 28 days (*p* = 0.008), also resulting in a significant difference with placebo‐treated hemifaces (*p* = 0.018).

**TABLE 1:** Normalized carbonyl levels of in vivo protein carbonylation.

Time	Placebo cream	AAE‐containing cream
Day 0	0.688 ± 0.255	0.670 ± 0.319
Day 28	0.669 ± 0.310	0.556 ± 0.216**/°

*Note*: Intra‐treatment statistical analysis versus D0: **p < 0.01. Inter‐treatment statistical analysis at a same timepoint: °*p* < 0.05.

## DISCUSSION

Extremophilic bacteria have developed elaborate strategies to survive in harsh environmental conditions. One such method relies on the synthesis of bacterioruberin, a C‐50 carotenoid. This compound has been implicated in the resistance to low temperatures or high salt concentrations, preservation of a functional membrane under different stresses, and as a potent antioxidant [29, 30, 31]. Compared with the nine pairs of double bonds present in β‐carotene, bacterioruberin has 13, which makes it a better ROS scavenger [38]. Despite its potentially beneficial properties, it remains largely unexplored [30].

Therefore, we analysed the ROS‐protective capacity of an AAE rich in bacterioruberins. As expected, AAE protected the in tubo alkaline phosphatase activity against oxidative stress. Even when tested at low concentrations, the level of protection achieved was similar to that of other antioxidants. This capacity of AAE to protect against oxidative stress is also evidenced in primary keratinocytes subjected to stresses that induce ROS production, such as organic pollutant‐coated PM, UVA, or blue light. In the present study, protein carbonylation, an early marker of protein oxidative stress [12, 13, 14, 15, 39], was drastically reduced when AAE was added to the culture medium. Even in ex vivo skin explants, pretreatment with a topical application of an AAE‐containing cream prevented protein carbonylation upon co‐exposure to UV and PM. The results from different skin layers indicated that tocopherol and AAE cream prevented epidermal protein carbonylation, whereas only AAE protected dermal proteins. Therefore, topically applied AAE can reach the dermis and exert its protective effects. Elastin quantification results further supported this outcome.

In addition to their potent antioxidant properties, bacterioruberins have been implicated in the protection against heat and salt stresses [32]. Thus, we evaluated whether AAE could protect proteins against these non‐oxidative stressors. In tubo, AAE protects alkaline phosphatase against high temperatures, also enabling progressive restoration of its activity and increasing the temperature at which BSA undergoes heat‐induced conformational change. Besides, AAE partially prevents elastin aggregation induced by a salt stress. Heat and salt stress resistance involves mechanisms different from those of oxidative stress, and antioxidants alone have no effect. This is exemplified by the powerful antioxidant Trolox. Considering the richness of AAE in bacterioruberins, it is likely that they are responsible for their protective effects. However, this protection did not depend on any carotenoid‐like mechanism of action. Phytoene, a C40 synthesis intermediate, exhibited no effect. In addition, considering the chemical nature of bacterioruberins and the concentrations evaluated, it is not possible for them to act as osmolytes that would equilibrate osmotic pressure and protect elastin upon salt stress, which is a frequent salt resistance mechanism in bacteria [40]. Therefore, the heat and salt stress resistance results demonstrate that bacterioruberins also have a different mode of action.

High salt concentrations affect proteins by stripping off the essential layers of surrounding water molecules and altering hydrogen bonds [41]. These hydrogen bonds are also affected by heat, owing to the extra energy. Both processes alter protein structure, solubility, and stability. Bacterioruberin partially prevents these alterations from occurring, suggesting that it physically interacts with proteins to protect them. In addition, it increases the temperature‐induced conformational change of BSA, as does 4‐phenyl butyrate, a well‐established chemical chaperone [35], indicating that both compounds should share a similar chaperone‐like activity. Like other small organic osmolytes, such as glycerol, glycine betaine, proline, trehalose, etc. – which also exhibit chaperone‐like properties [42, 43, 44, 45, 46, 47], bacterioruberin appears to nonspecifically stabilize native protein structures [48, 49, 50], protecting proteins from denaturation and aggregation, and assisting the functional recovery of proteins following stress [23, 24, 25, 26].

Importantly, AAE‐induced protection was also observed in the facial skin of smokers. Cigarette smoke generates various organic pollutants, leading to oxidative stress that affects all skin layers [51]. After 28 days of application of AAE‐containing cream, the level of carbonylated protein was reduced in the upper *stratum corneum* cell layers sampled, showing that AAE protects against oxidative stress induced by the environment, including cigarette smoke. However, terminally differentiated keratinocytes, the corneocytes that compose the *stratum corneum*, remain there for ~ 14 days before desquamation [52]. Deprived of a nucleus and essentially consisting of keratin, corneocytes show no protein synthesis. This implies that AAE protected the corneocyte protein content during these 14 days. In addition, the epidermal turnover time, which is the time needed for a keratinocyte to leave the basal membrane and turn into a desquamating corneocyte, is ~ 45 days [53]. Considering that the skin explant experiment showed penetration of AAE into deep skin layers, the reduced carbonylation level evidenced in vivo also indicates that AAE probably protected proteins at earlier time points, when keratinocytes were still active, undergoing their terminal differentiation into corneocytes, and having active protein synthesis. This experimental series also showed the positive influence of AAE on the dermis. Therefore, it is likely that topical application of AAE‐containing creams protects the dermal proteome in vivo. Further studies, including more invasive methods, such as punch biopsies, should provide deeper insights into the in vivo beneficial effects of AAE. Nevertheless, the topical application of antioxidants leads to visible antiageing effects [54, 55], and it is reasonable to assume that an AAE‐containing cream would have similar benefits. Considering the high antioxidative and protective activities of the extract owing to its peculiar dual mode of action, AAE is expected to have a potent effect on ageing signs. Future studies should validate this.

## CONCLUSIONS

Regardless of the exact mechanism(s) by which AAE acts and its properties, its antioxidative and chaperone‐like activities result in a two‐pronged mechanism, which explains its high capacity to protect the proteome against various stressors, especially oxidative stress. By contributing to the maintenance of natural resources and mechanisms of the skin, AAE helps to preserve the equilibrium of the ever‐evolving skin ecosystem and participates in its ecobiological protection [10, 27]. However, considering the importance of oxidative stress in the onset of diseases [56], exploring the potential of AAE in disease prevention would also be interesting. Ad hoc clinical studies should aim to elucidate this aspect.

## FUNDING INFORMATION

This work was funded by NAOS.

## CONFLICT OF INTEREST STATEMENT

JCT, FXP, NL, AF, FG, EP ST and IB are or were employees of NAOS.

## Data Availability

The data that support the findings of this study are available from the corresponding author upon reasonable request.
